# Olfactory dysfunction in Alzheimer’s disease Systematic review and meta-analysis

**DOI:** 10.1590/1980-57642018dn12-020004

**Published:** 2018

**Authors:** Maren de Moraes e Silva, Pilar Bueno Siqueira Mercer, Maria Carolina Zavagna Witt, Renata Ramina Pessoa

**Affiliations:** 1 MD. Hospital da Cruz Vermelha Filial do Paraná. Neurology Department. Curitiba, PR, Brazil.

**Keywords:** Alzheimer’s disease, olfactory disorders, dementia, early diagnosis, doença de Alzheimer, transtornos do olfato, demência, diagnóstico precoce

## Abstract

Alzheimer’s disease (AD), a neurodegenerative condition, is one of the most prevalent kinds of dementia, whose frequency doubles for every 5 years of age in elderly.

**Objective:**

To determine the correlation between AD and olfactory alterations, identifying the most affected domains and exploring the utility of olfactory tests for complementing early diagnosis.

**Methods:**

Databases were searched using the terms “olfactory OR smell OR olfaction AND alzheimer” for articles related to the proposed theme. The selected studies were categorized and evaluated separately depending on the method of analysis of the olfactory tests: identification of odors, discrimination and recognition, and a meta-analysis was carried out.

**Results:**

Fifty-one articles were selected for analysis. The effect size for most studies was large, as were the summary values for each category of individualized olfactory analysis.

**Conclusion:**

Among the olfactory domains, except memory, identification appears to be the most altered in AD. The possibility of including tests that specifically evaluate the identification of odors as an item in early diagnostic evaluation should be explored. PROSPERO registration: CRD42018089076.

## Resumo

Neurodegenerativa, a doença de Alzheimer (DA) é um dos tipos mais prevalentes de demência, com frequência dobrando a cada 5 anos em idosos.

**Objetivo:**

Verificar a correlação entre DA e alterações olfatórias, identificando os domínios mais afetados e a possibilidade de utilização de testes olfatórios como complemento de diagnóstico precoce.

**Métodos:**

Bases de dados foram acessadas utilizando os termos “olfactory OR smell OR olfaction AND alzheimer” buscando artigos relacionados ao tema proposto. Os estudos selecionados foram categorizados e avaliados em separado a depender do método de análise olfatória: identificação de odores, discriminação e reconhecimento e uma meta-análise foi realizada.

**Resultados:**

Cinquenta e um artigos foram selecionado para análise. O tamanho do efeito da maioria dos estudos foi grande, assim como os valores sumários de cada categoria de análise olfatória.

**Conclusão:**

Entre os domínios olfatórios, excetuando memória, a identificação parece ser a mais alterada em DA. É possível explorar a possibilidade de adição de testes específico para avaliação de identificação de odores como um item na avaliação diagnóstica precoce. Registro PROSPERO: CRD42018089076.

Alzheimer’s disease (AD), a neurodegenerative condition, is one of the most prevalent kinds of dementia, whose frequency doubles for every 5 years of age in elderly.^1^ Although the spectrum of the disease is more often related to cognitive disorders, it is necessary to pay attention to other factors related to the process of illness for global analysis of the patient and, farther, as a means of seeking additional methods of early diagnosis. Currently, olfaction seems to be one of these factors. This basic sense is affected with normal aging,^2^ however, it seems to be even more intensely impaired in patients with different neurodegenerative diseases,^3^ including AD, bearing in mind that olfaction is also correlated with recall mechanisms due to its synchronization with the hippocampus in the process of creation and retrieval of olfactory associative memory.^4^ In addition, a possible change in components of g- secretase enzymes has been reported in previous studies as suggestive of olfactory alterations in patients with recent-onset AD,^5^ besides an influence of tau protein deposits in the olfactory bulb affecting the limbic system directly - a fact that was highly evident in AD and less frequently in healthy individuals.^6^ This highlights the importance of the study of these mechanisms to clarify the pathophysiology of degenerative diseases that affect the central nervous system.

A systematic review by Rahayel et al.^7^ previously evaluated the correlation between Alzheimer’s disease and olfaction compared to Parkinson’s disease, analyzing studies dated up to 2010, however, a lot of new material has been published addressing this topic in the last 8 years, calling for an updated analysis. The present study aimed to determine the correlation between AD and olfactory alterations, identifying the most affected domains and exploring the utility of olfactory tests for complementing early diagnosis.

## METHODS

This is a systematic review and meta-analysis, registered on the PROSPERO database, under register CRD42018089076.

### Study eligibility

Prior to the systematic search, a brief database search was carried out to identify possible key words to guide the review.

To search for articles to be included in the review, the databases MEDLINE - PubMed, SciELO and LILACS were used with the keywords “olfactory OR smell OR olfaction AND alzheimer”. We selected studies investigating olfactory function in patients diagnosed with Alzheimer’s disease compared to age-matched healthy controls. Inclusion criteria were articles in Portuguese and English, conducted in an adult population, and without publication date restriction. Cross-sectional and longitudinal, retrospective and prospective observational studies were included, whereas editorials, guidelines, letters and reviews were excluded. Exclusion criteria were also studies that did not describe the method used for diagnosing Alzheimer’s disease, those that did not have a control group with age-matched individuals, and papers that did not provide the necessary information for performing the meta-analytical statistical analysis, even after contacting the respective authors, and whose values could not be calculated by us based on the data given in the articles. The literature search was concluded in 2018 February.

### Selection process

The selection was initially done by reviewing titles and abstracts matching the inclusion criteria, together with an assessment based on the “PICO”^8^ strategy, providing initial screening of the potentially eligible studies. Based on this acronym-tool, “P” refers to “participants” (in our case, research in adult humans with Alzheimer’s disease), “I” refers to “intervention” (olfactory alteration screening instruments), “C” for “comparison” (adult patients without dementia) and “O” to “outcomes” (correlation of olfactory impairment with the presence or absence of dementia). After the initial selection, the papers were read in full, excluding those that did not fit the study inclusion/exclusion criteria outlined previously. All of the processes described above were performed by two independent researchers. Disagreements were resolved by consensus. Finally, the kappa value was calculated as a means of evaluating the agreement level for the eligibility of the studies.

### Data analysis

As a way of standardizing the results of the analyzed tests, Cohen’s D^9^ was used to calculate effect size. Results <0.2 were considered as a low effect, >0.5 as medium and ≥0.8 as having a large effect.

The homogeneity of the included studies was analyzed by Cochran Q^10^ and I^2^ statistics. In cases presented as heterogeneous, the data were reassessed using meta-regression techniques and by subgroup analysis, when appropriate. Continuous variables were analyzed by the Mann Whitney test and Pearson’s correlation test. The Kruskal Wallis test was also used to compare the three groups.

Statistical analysis was performed using R^11^ software and forest plots were generated by “DistillerSR Forest Plot Generator tool from Evidence Partners”, available online.^12^ Necessary information for statistical analysis that was not explicit in the article itself was calculated based on published data and, when not possible to be obtained in any other way, were requested from the authors by electronic mail.

The publication bias was assessed by creating a funnel plot for subsequent analysis according to Duval and Tweedie (“trim and fill”)^13^ and Roshental (“Fail safe N”) methods.^14^

### Study categorization

The selected studies were categorized and evaluated separately depending on the method of analysis of the olfactory tests: identification of odors, discrimination and recognition. Identification is understood here as the ability to name the smell, while discrimination refers to the ability to detect specific olfactory stimulus or evaluation of the olfactory threshold in a series of tests, and “recognition” means the study of olfactory memory.

Articles that analyzed more than one of these domains underwent separate statistical analysis, according to their categorization.

## RESULTS

### Eligible study selection

**Figure 1:**
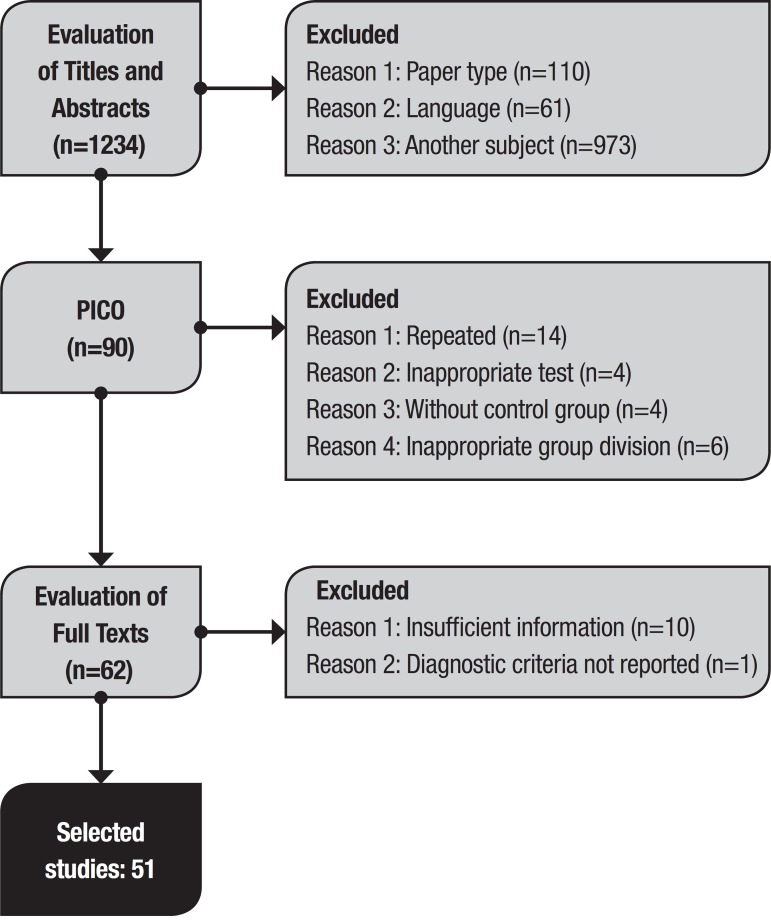
Study selection flowchart.

The search of the literature using the key terms retrieved a total of 1234 articles. Of these, 1144 were excluded after the initial review of titles and abstracts. Of the remaining articles, 28 were excluded after applying the PICO criteria and, after access to the full text, 11 articles were withdrawn. This selection sequence is depicted in figure 1 along with the reasons for exclusions. Authors’ concordance was calculated with a kappa of 0.95.

### Characteristics of selected studies

The selected studies encompassed papers published over the last 32 years: the oldest from 1986 and the most recent dating to 2017. Most of these studies were from the United States of America, followed by European countries in conjunction. No studies published by South American or African countries were found.

In relation to categorical stratification, the identification of odors was measured 40 times,^15^^-^ 52 discrimination 21 times^16^^,^ 21 ^,^ 22 ^,^ 26 ^,^ 29 ^,^ 34 ^,^ 35 ^,^ 37 ^,^ 38 ^,^ 46 ^,^ 53 ^-^ 61 and recognition only 6 times,^17^^,^ 18 ^,^ 40 ^,^ 57 ^,^ 62 ^,^ 63 representing samples of 3328, 1062 and 244 evaluated individuals, respectively.

Individual characteristics of selected articles are given in Table 1.

**Table 1:** Study characteristics.

Author (Year)	Country	Modality	Tool	Criteria
Warner, et al (1986)	USA	Identification	UPSIT-40	DSM III
Rezek, et al (1987)	USA	Identification	Homegrown	Berg et al
Moberg, et al (1987)	USA	Recognition	Homegrown	DSM III
Kesslak, et al (1988) [a]	USA	Identification	UPSIT-40	NINCDS-ADRDA
Kesslak, et al (1988) [b]	USA	Recognition	UPSIT-40	NINCDS-ADRDA
Kesslak, et al (1991) [a]	USA	Identification	UPSIT-40	NINCDS-ADRDA
Kesslak, et al (1991) [b]	USA	Recognition	UPSIT-40	NINCDS-ADRDA
Serby, et al (1991)	USA	Identification	UPSIT-40	NINCDS-ADRDA
Buchsbaum, et al (1991)	USA	Recognition	Match-to-sample test	NINCDS-ADRDA
Nordin, et al (1995)	USA	Discrimination	Homegrown	NINCDS-ADRDA and DSM III-R
Moberg, et al (1997)	USA	Identification	UPSIT-40	NINCDS-ADRDA
Larsson, et al (1999) [a]	Sweden	Identification	Homegrown	NINCDS-ADRDA
Larsson, et al (1999) [b]	Sweden	Discrimination	Homegrown	NINCDS-ADRDA
Kareken, et al (2001) [a]	USA	Identification	UPSIT-40	NINCDS-ADRDA
Kareken, et al (2001) [b]	USA	Discrimination	Homegrown	NINCDS-ADRDA
Royet, et al (2001)	France	Identification	Homegrown	NINCDS-ADRDA
Chan, et al (2002)	China	Identification	Homegrown	DSM IV
Duff, et al (2002)	USA	Identification	PST	DSM IV
Peters, et al (2003) [a]	Germany	Identification	SS-OIT	NINCDS-ADRDA
Peters, et al (2003) [b]	Germany	Discrimination	SS-OIT	NINCDS-ADRDA
Getchell, et al (2003)	USA	Discrimination	Homegrown	NINCDS-ADRDA
Suzuki, et al (2004)	Japan	Identification	CC-SIT	DSM IV and NINCDS-ADRDA
Gilbert, et al (2004-1) [a]	USA	Discrimination	Homegrown	NIA, CERAD, DSM III and NINCDS-ADRDA
Gilbert, et al (2004-1) [b]	USA	Recognition	Homegrown	NIA, CERAD, DSMIII and NINCDS-ADRDA
Gilbert, et al (2004-2)	USA	Discrimination	Homegrown	NIA and CERAD
Tabert, et al (2005) [a]	USA	Identification	UPSIT-40	DSM IV
Tabert, et al (2005) [b]	USA	Identification	B-SIT	DSM IV
Tabert, et al (2005) [c]	USA	Identification	10-item Scale	DSM IV
Djordjevic, et al (2006) [a]	Canada	Identification	UPSIT-40	NINCDS-ADRDA
Djordjevic, et al (2006) [b]	Canada	Discrimination	Homegrown	NINCDS-ADRDA
Kjelvik, et al (2007)	Norway	Identification	B-SIT	NINCDS-ADRDA
Pentzek, et al (2007)	Germany	Identification	SS-OIT	NINCDS-ADRDA
McLaughlin, et al (2007)	USA	Identification	B-SIT	NINCDS-ADRDA
Sundermann, et al (2007)	USA	Discrimination	Homegrown	NINCDS-ADRDA and DSM III - R
Jungwirth, et al (2009)	Austria	Identification	PST	NINCDS-ADRDA
Steinbach, et al (2009)	Germany	Identification	SS-OIT	NINCDS-ADRDA
Williams, et al (2009) [a]	United Kingdom	Identification	SS-OIT	NINCDS-ADRDA
Williams, et al (2009) [b]	United Kingdom	Discrimination	SS-OIT	NINCDS-ADRDA
Steinbach, et al (2009)	Germany	Discrimination	SS-OIT	NINCDS-ADRDA
Förster, et al (2010)	Germany	Identification	SS-OIT	NINCDS-ADRDA
Li, et al (2010) [a]	USA	Identification	UPSIT-40	NINCDS-ADRDA
Li, et al (2010) [b]	USA	Discrimination	UPSIT-40	NINCDS-ADRDA
Razani, et al (2010)	USA	Identification	Homegrown	NINCDS-ADRDA and DSM III-R
Wang, et al (2010)	USA	Identification	UPSIT-40	NINCDS-ADRDA
Bahar-Fuchs, et al (2010) [a]	Australia	Identification	UPSIT-10	NINCDS-ADRDA
Bahar-Fuchs, et al (2010) [b]	Australia	Recognition	Homegrown	NINCDS-ADRDA
Razani, et al (2010)	USA	Discrimination	Homegrown	NINCDS-ADRDA and DSM III-R
Bahar-Fuchs, et al (2011)	Australia	Identification	UPSIT-6	NINCDS-ADRDA
Makowska, et al (2011)	Poland	Identification	PST	NINCDS-ADRDA
Schofield, et al (2012)	Australia	Identification	UPSIT-20	DSM IV and NINCDS-ADRDA
Velayudhan, et al (2013)	United Kingdom	Identification	UPSIT-40	NINCDS-ADRDA
Seligman, et al (2013)	USA	Identification	SS-OIT	CERAD
Servello, et al (2015) [a]	Italy	Identification	SSET	NINCDS-ADRDA
Servello, et al (2015) [b]	Italy	Discrimination	SSET	NINCDS-ADRDA
Velayudhan, et al (2015)	United Kingdom	Identification	UPSIT-40	NINCDS-ADRDA
Hori, et al (2015)	Japan	Discrimination	Homegrown	NINCDS-ADRDA and DSM IV
Vyhnalek, et al (2015)	Czech Republic	Discrimination	MHST	NINCDS-ADRDA and DSM IV
Hagemeier, et al (2016)	USA	Identification	UPSIT-40	NINCDS-ADRDA
Passler, et al (2016)	USA	Identification	UPSIT-40	ICD-9-CM
Reijs, et al (2017)	Europe	Identification	B-SIT	NINCDS-ADRDA
Christensen, et al (2017)	Denmark	Identification	PST	Gauthier, 2006
Quarmley, et al (2017)	USA	Identification	SS-OIT	CERAD

UPSIT-40: University of Pennsylvania Smell Identification 40 items; DSM III: Diagnostic and Statistical Manual of Mental Disorders III Edition; NINCDS-ADRDA: National Institute of Neurological and Communicative Disorders and Stroke - Alzheimer’s Disease and Related Disorders Association; DSM III-R: Diagnostic and Statistical Manual of Mental Disorders III Edition Revised; DSM IV: Diagnostic and Statistical Manual of Mental Disorders IV Edition; PST: Pocket Smell Test; SS-OIT: Sniffin’ Sticks Odor Identification Test; CC-SIT: Cross Cultural Smell Identification Test; NIA: National Institute on Aging; CERAD: Consortium to Establish a Registry for Alzheimer’s Disease; B-SIT: Brief Smell Identification Test; UPSIT-10: University of Pennsylvania Smell Identification 10 items; UPSIT-6: University of Pennsylvania Smell Identification 6 items; UPSIT-20: University of Pennsylvania Smell Identification 20 items; SSET: Sniffin’ Sticks Extended Test; MHST: Motol Hospital Smell Test.

### Olfactory evaluation tools

Eleven tools for assessing olfactory ability were evaluated (with all “homegrown” instruments considered as only 1 type, regardless of the particularity of each). The most commonly used included the University of Pennsylvania Smell Identification Test (UPSIT) and the Sniffin’ Sticks Odor Identification Test (SSOIT).

The UPSIT tool (produced by Sensonics Inc., Haddon Heights, NJ) consists of 40 types of odors associated with multiple choice questions. Some studies included in the present review used this scale partially as a means of testing the applicability of a faster scale.

The SSOIT test entails presenting only 16 odors to the patient, who also answers multiple-choice questions with 4 items each.

### Demographic characteristics

The mean age of the patients evaluated in the Alzheimer’s disease groups for identification tests was 73.52 years (95%CI 72.32-74.72, SD 3.81), ranging from 64.2 to 81.9 years old, whereas the control group had a mean age of 70.85 years (95%CI 68.68-73.02, SD 6.88), ranging from 63.4 to 79.6 years. For the same category, the gender ratio was predominantly female, with a 1.4:1 proportion. The correlations between age and sex predominance in relation to olfactory performance were not statistically significant (R 0.004 p 0.98 and Z -0.27 p 0.79, respectively).

The mean Mini-Mental State Examination (MMSE) score was 21.61 (95%CI 20.89-22.33, SD 1.96), with a mean educational level of 11.94 years of study (95%CI 11.07-12.81, SD 2.06) among the identification studies. All articles evaluated individuals with an average of 9 or more years of education, with only the paper by Chan et al.^24^ below this level, with an average of 5 years. The correlation between MMSE performance and olfactory tests was not statistically significant (R -0.35, p 0.059).

### Effect size and group comparison

The effect size for most studies was large, as were the summary values ​for each category of individualized olfactory analysis. For identification, the value of d ranged from 0.37 to 5.65 (mean 1.99, 95% CI 1.59-1.66), for discrimination from 0.28 to 2.76 (mean 0.81, 95% CI 0.77-0.85), and recognition from 0.38 to 8.33 (mean 3.13, 95% CI 1.15-1.86).

**Figure 2:**
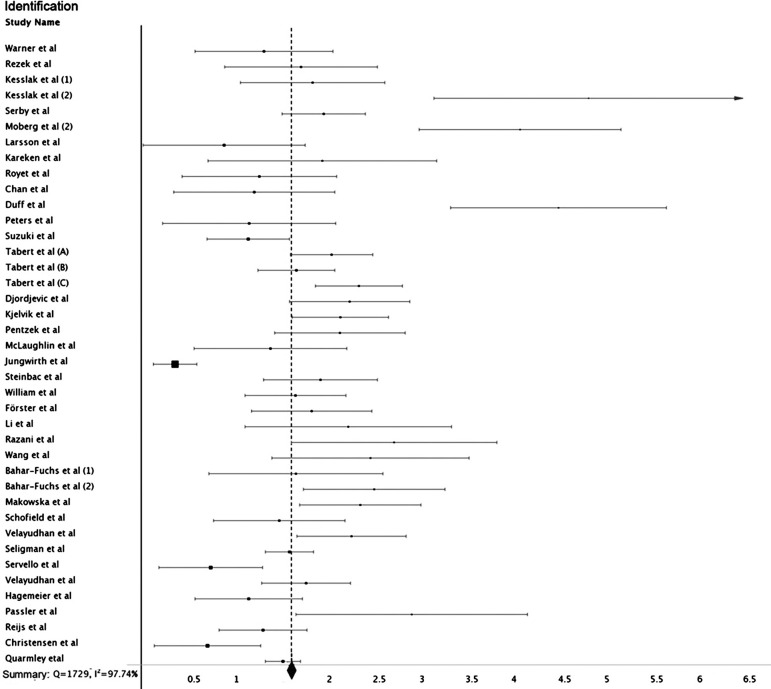
Individual study effect sizes: identification domain.

**Figure 3:**
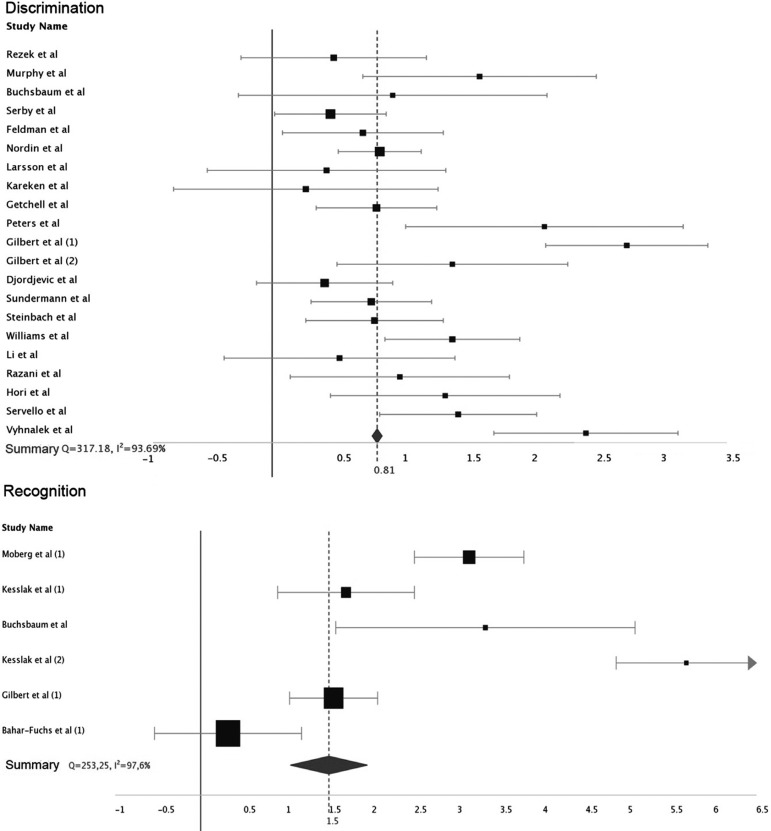
Individual study effect sizes: discrimination and recognition domains.

All categories were heterogeneous in analysis, with Q values ​of 253.25 (I^2^ 97%), 317.18 (I^2^ 93%) and 1729 (I^2^ 97%) for recognition, discrimination and identification, respectively (Figures 2 and 3).

The difference between the three olfactory domains was statistically significant (H 14.51 df 2 p<0.001), with the difference between identification and discrimination being more intensely significant (Z 3.65 p<0.001). There were too few studies addressing recognition for accurate statistical analysis in terms of comparison.

Statistical differences between the UPSIT (mean d 1.18) and SSOIT (mean d 1.6) tests were also analyzed separately, with a statistically significant relationship (Z 2.48 p 0.012).

### Moderator analysis

The evaluation of the possible factors of heterogeneity through meta-regression revealed that heterogeneity was maintained despite the use of moderators: MMSE (k=30 p=0.59 Z= -0.53). age k=40 p=0.98 Z= -0.02). sample size (k=40 p=0.61 Z= -0.50) and gender (k= 37 p=0.90 Z=0.12).

Analysis in relation to smoking could not be performed due to insufficient information: only 9 studies^19^^,^ 22 ^,^ 26 ^,^ 27 ^,^ 36 ^,^ 42 ^,^ 44 ^,^ 46 ^,^ 47 employed this item as an exclusion criterion and only 5 articles^20^^,^ 21 ^,^ 25 ^,^ 29 ^,^ 34 reported the number of smokers present in the sample. The same situation occurred for onset and duration of symptoms of AD, with insufficient data available. Most studies defined history of prior brain injury as an exclusion criteria. However, when the studies that did not take this criteria into account or did not report this detail were removed, the heterogeneity was maintained (k=30, p<0.0001, z-11.58).

Regarding the type of tool for evaluation, a subgroup analysis was carried out based on the categories UPSIT, SSOIT and “others”: the heterogeneity between the studies persisted. Analysis by diagnostic criteria subgroups also maintained heterogeneity.

### Publication bias

Visual analysis of the funnel plot showed asymmetry. Subsequently, the application of Duval and Tweedie^13^ method revealed the need for 21 studies on the right side to ensure symmetry. However, the analysis using the Roshental approach^14^ revealed that 18347729 “null” studies would need to be incorporated into the present review to negate the effect observed here (p<0.001).

## DISCUSSION

Correlation between olfactory changes and neurodegenerative diseases has been extensively analyzed in current medical literature. The present meta-analysis aimed to specifically evaluate these alterations in Alzheimer’s disease, which is associated with impairment both in terms of identification and discrimination of odors, as well as recognition. This fact was corroborated by the present study, which found a large effect size. These findings are in agreement with previous analyses of a smaller number of studies,^7^ including in the case of early diagnosis, and are in line with a previous meta-analysis of these alterations in mild cognitive impairment.^64^

Among the olfactory domains, except memory, identification appears to be the most altered in AD, in agreement with previous studies.^7^ It is also necessary to take into account the fact that, even in healthy elderly groups there is a reduction in olfactory sensitivity.^2^ However, domains are affected differently in relation to Alzheimer’s disease, as can be verified in analyses demonstrating that in the healthy elderly population discrimination (olfactory thresholds) is the most affected.^2^ These differences can be explained by the association of execution and memory cognitive domains, related in part to performance on tests that involve identification and recognition, being closely related with semantic memory^65^ - factors which should be taken into account when choosing the best test for olfactory evaluation.

On the other hand, differences in relation to gender proportion of samples among the elderly population can be expected, since women have higher life expectancies.^66^ However, the present study failed to find a statistical difference between the genders. By contrast, a previous study by Roalf et al.^64^ involving a cognitive impairment analysis, found a significant difference between genders, showing greater involvement in men.

An additional point to be analyzed and which required separate evaluations, were the changes in the current diagnostic criteria, considering that the analyzed studies were published over a 30-year period and that the diagnostic criteria used may have differed.^67^^,^ 68 However, these modifications do not appear to have had a substantial impact on the results when a meta-regression was performed.

Regarding correlation between MMSE score and olfactory evaluation, it is surprising that no statistically significant difference was found. This is possibly due to the small sample size adopted by many of the studies included in this review, which may have greatly impaired the analysis. In view of the previously explained relationship between certain types of olfactory assessment and cognitive issues, a significant difference could be expected between the disease and control groups. However, this finding is in agreement with previous studies evaluating pre-morbid conditions.^64^ Conversely, an observational study found a correlation between low score in cognitive screening and larger olfactory sensory deficits.^40^ Given this evidence, it is prudent that in dementia, the actual sensory alteration measured by the tests is differentiated from cognitive alterations, for adequate analysis of predictive value for initial disease and/or worse outcome. An alternative hypothesis for non-correlation may be the fact that the MMSE provides only a superficial study, requiring a complete and extensive neuropsychological evaluation to differentiate the origin of the observed deficits.

Several types of tests for distinguishing olfactory deficits can be used, while many studies have devised their own tests, demonstrating their ease of execution. On the other hand, this heterogeneity of methods can hamper global statistical analysis. The most commonly used commercial tests include UPSIT and SSOIT, with a statistically significant difference between them in the present study. Results indicated a greater effect for the SS-OIT, possibly due to the shorter application time, requiring less effort for the patient. When using both tests, we are again facing the question of cognitive-sensory differentiation, since both use forced multiple-choice questions. The use of other types of tools and approaches can help elucidate and differentiate the aspects affected by the disease.

This review is limited in relation to the risk of publication bias, as explained in the results. In addition, it is important to mention that the vast majority of the studies that addressed the application of the tests had samples containing individuals with medium-to-high education from developed countries. Therefore, care should be taken when generalizing these results to populations with different educational levels and cultural backgrounds.

The presence of greater olfactory involvement in patients with AD is clear, and the possibility of including tests that specifically evaluate the identification of odors as an item in early diagnostic evaluation, and maybe prognosis, together with a detailed cognitive evaluation, should be explored.

Further studies are needed to evaluate this applicability in other populations, taking a more homogeneous methodological approach in terms of gender distribution and assessing confounding factors such as previous smoking.
